# 

**Published:** 1954

**Authors:** 


### Contents

| | | Page |
|---|---|---|
| EDITORIAL | | 31 |
| MORTIMER HOUSE, CLIFTON<br>MISS NOTT OF ST. BRENDA'S<br>CASUALTY OFFICERS<br>NEUROSURGICAL UNIT, FRENCHAY | | |
| OBSERVATIONS ON BLINDNESS | *Mr. R. Ramsay Garden* | 33 |
| DIFFUSE ANGIOMATOSIS OF THE SMALL INTESTINE CAUSING MELAENA | *Mr. John A. Vere Nicoll* | 47 |
| PHAEOCHROMOCYTOMA OF ADRENAL GLAND WITH PAROXYSMAL HYPERTENSION | *Dr. G. M. Colson* | 50 |
| HERPES ZOSTER ENCEPHALITIS | *Dr. R. D. G. Creery* | 54 |
| HERPES ZOSTER GENERALISATUS | *Dr. Mark Hewitt* | 57 |
| CORRESPONDENCE | *Dr. D. H. Forster* | 61 |
| NEWS FROM SOCIETIES | | 62 |
| BRISTOL MEDICO-CHIRURGICAL SOCIETY<br>MALIGNANT DISEASE: THE PROBLEM OF THE ADVANCED CASE | | |
| APPOINTMENTS | | 65 |
| NOTES AND NEWS | | 67 |
| POSTGRADUATE COURSES FOR GENERAL PRACTITIONERS<br>MEDICAL EXHIBITION<br>WESSEX RAHERE CLUB | | |

